# Hypothetical e-liquid flavor ban and opinions among vape shop retailers in the Greater Los Angeles Area

**DOI:** 10.18332/tid/172078

**Published:** 2023-10-13

**Authors:** Sabrina L. Smiley, Heesung Shin, Nichelle Brown, Angela A. Geraci, Steve Sussman

**Affiliations:** 1 Division of Health Promotion and Behavioral Science, School of Public Health, College of Health and Human Services, San Diego State University, San Diego, United States; 2 Department of Population and Public Health Sciences, Keck School of Medicine, University of Southern California, Los Angeles, United States

**Keywords:** ban, e-liquid, flavor, vape shops

## Abstract

**INTRODUCTION:**

Evaluating anticipated responses to flavor bans in the context of vape shops is needed to inform legislation and enforcement. This cross-sectional study examined vape shop retailers’ opinions about the potential impacts of an e-liquid flavor ban on shop sales and customer behavior-change intentions.

**METHODS:**

From December 2019 to October 2020 we conducted structured interviews over the phone with 46 brick-and-mortar vape shop retailers in the Greater Los Angeles Area.

**RESULTS:**

Most participants were managers (43.5%), followed by owners (26.1%) and clerks (26.1%). More than half (52.2%) reported that sales would drop a lot if flavored e-liquids were banned in all vape shops. Controlling for store position, multivariable linear regression showed that opposition to a hypothetical ban on non-tobacco flavored e-liquids was associated with participants’ opinions that customers would likely not purchase tobacco flavored e-liquids (b= -0.44, p<0.01), and would likely use combustible tobacco products (b=0.47, p<0.05).

**CONCLUSIONS:**

In this cross-sectional study, vape shop retailers in the Greater Los Angeles Area reported that if a ban on non-tobacco e-liquid flavors occurred, they would oppose strongly, and that a ban would have a negative impact on their shop (e.g. loss in sales) and customer behavior (e.g. would replace vaping with smoking combustible tobacco products). Implications for research and practice are discussed.

## INTRODUCTION

Sales and use of electronic nicotine delivery systems (ENDS) have continued to significantly increase in the US in recent years^1,2^. One of the most important reasons for the rapid increase in ENDS use is the large variety of available e-liquid flavors (e.g. candy, fruit, menthol, tobacco)^3,4^. Flavors play an important role in the initiation and use of ENDS among US youth and adults^5-7^. Research has demonstrated that for most ENDS users, the first and current e-liquid had a non-tobacco flavor^8-10^.

With the large variety of appealing flavors, there have been pivotal changes in the US nicotine and commercial tobacco retail landscape. In response to the surge in ENDS use among youth, in February 2020, the US Food and Drug Administration (FDA) restricted the manufacture, distribution, marketing, and sale of prefilled cartridge-based ENDS (e.g. JUUL) in flavors other than menthol and tobacco^11^. Additionally, many US states and localities have been implementing or proposing policies with various flavor restrictions. For example, a ‘yes’ vote in November 2022 upheld Senate Bill 793^12^, which prohibits the sale of flavored nicotine and commercial tobacco products in California, with exception of premium cigars and hookah tobacco. Given the policy changes at the federal/state/local level, there is a critical need from an applied research perspective to understand how stakeholders perceive e-liquid flavors, which is the focus of this study.

Vape shop retailers are a relevant stakeholder group given that they directly interact with different types of ENDS users^13^. In order for regulatory policymaking to maximize effectiveness, the perceptions and behaviors of retailers in promoting and selling ENDS must be considered. Previous research with licensed commercial tobacco retailers in a jurisdiction with a local flavor ordinance (e.g. Boston, Massachusetts, San Franscisco, California) found that not knowing whether certain nicotine and commercial tobacco products were flavored was a major concern for retailers^14,15^. Prior to Senate Bill 793^12^, research with licensed commercial tobacco retailers in the Greater Los Angeles Area found that perceptions of ENDS, as being completely safe/safer than cigarettes, were significantly associated with availability of flavored e-cigarettes^16^. A recent opinion poll with licensed commercial tobacco retailers in California found that retailers in a jurisdiction with a flavor ordinance expressed lower support for flavor sales restrictions compared to retailers in a jurisdiction without a flavor ordinance^17^.

Given the instrumental role that retailers play in the vape industry, it is important to understand their perceptions of vape product regulations. Policies prohibiting the sale of e-liquid flavors may influence a retailer’s perception, thereby leading to intention to change and actual change in behaviors. Nevertheless, evaluating the potential impacts of such policies among vape shop retailers has remained understudied. This study fills a gap in the existing literature by examining opinions about the potential impacts of an e-liquid flavor ban on shop sales and customer behavior-change intentions among a sample of vape shop retailers in the Greater Los Angeles Area.

## METHODS

### Participants and procedures

A Yelp search was conducted to identify brick-and-mortar vape shops in the Greater Los Angeles Area. We excluded stores considered commercial tobacco shops or other stores (i.e. convenience stores) that sold items unrelated to vaping (e.g. combustible tobacco products). The sampling frame of the vape shops assessed is detailed elsewhere^18^. A total of 61 vape shops identified through a Yelp search were contacted via phone call to assess/confirm willingness to participate and to schedule an interview. A total of 46 vape shops consented (when called to complete/schedule the interview) and participated in the current study.

### Data collection

Data were collected in December 2019 to October 2020 from brick-and-mortar vape shops in the Greater Los Angeles Area. Data collection methods included interviews with either the owner or an employee (hereon referred to as retailers) based on availability. Although the current study focused on vape shop retailers’ opinions about the potential impacts of an e-liquid flavor ban, it is important to note that during data collection, the FDA implemented on 6 February 2020, an ENDS flavor enforcement policy to restrict the manufacture, distribution, marketing, and sale of prefilled cartridge-based ENDS (e.g. JUUL) in flavors other than menthol and tobacco^11^. The study methods were approved by the University of Southern California Institutional Review Board (IRB00097895). All interviews (about 20 minutes duration) were conducted verbally by trained project staff. The retailers who participated in the interview received a $50 gift card for their time (one interviewee per shop).

### Measures

The interviewer asked participants about their age, sex, race/ethnicity, and position at the vape shop (i.e. owner, manager, clerk, or other). Those who answered ‘other’ were further asked to state their position at the vape shop. Self-reported measures of customer demographics (i.e. sex, age, race/ethnicity) were also assessed by the interviewer, and response categories were closed-ended. The number of flavored e-liquids available at the vape shop was assessed by asking participants the question: ‘How many e-juice or e-liquid flavors do you sell at this shop?’. Responses were open-ended and included a ‘don’t know’ category. Opinions about rules banning non-tobacco flavored e-liquids were measured using the question: ‘What’s your opinion on rules such that only tobacco-flavored e-juices or e-liquids were allowed at all vape shops?’. Response categories included: ‘favor strongly’, ‘favor somewhat’, ‘oppose somewhat’, and ‘oppose strongly’. Opinions about potential effects of a ban on flavored e-liquids on their shop were measured using the question: ‘How much do you think your sales would drop if there was a ban on flavored e-juice or e-liquid?’. Response categories included: ‘none’, ‘a little’, ‘half’, ‘a lot’, and ‘all’. Opinions about potential effects of a ban on flavored e-liquids on customer behavior-change intentions were measured using two questions: ‘How likely is it that customers would continue to purchase vape products and e-juices if rules such that only tobacco-flavored e-juices or e-liquids were allowed at all vape shops?’ and ‘How likely is it that customers would use smokable tobacco products if rules such that only tobacco-flavored e-juices or e-liquids were allowed at all vape shops?’. Response categories included: ‘not at all’, ‘a little’, ‘moderately’, and ‘extremely’.

### Data analysis

Descriptive statistics were calculated for participant demographic characteristics and self-reported measures of customer demographics. Three separate multivariable linear regression models were conducted to assess the relationship between opinions on rules prohibiting the sale of non-tobacco flavored e-liquids and the perceived impact on their shop and customer behavior-change intentions: Model 1 – purchase tobacco-flavored e-liquids; Model 2 – continue to vape; and Model 3 – use smokeable tobacco. All models controlled for participant shop position, using the ‘other’ shop position category as the reference. All variables included in the model as predictors or outcomes were used as continuous variables without re-coding. Analyses were conducted using SAS software, Version 9.3 (SAS Institute, Cary NC).

## RESULTS

### Vape shop retailer demographic characteristics and descriptions of their customers

Participants interviewed were predominantly male (87%), with average age 31.9 years (SD=8.5). Most participants were managers (43.5%), followed by owners (26.1%), clerks (26.1%), and those who answered ‘other’ were cashiers (4.3%). Participants primarily self-identified as Hispanic/Latino (28%), non-Hispanic White (28%), or Korean American (24%). Participants described their customers as being male (58.7%), young adult (aged 20–30 years; 47.8%), non-Hispanic White (28%), and Hispanic/Latino (24%).

### Vape shop retailers’ perceived impact of a hypothetical ban on non-tobacco flavored e-liquids

Most participants (87%) reported strongly opposing a ban on non-tobacco e-liquid flavors if it was enforced. Most (87%) reported selling more than 50 e-liquid flavors at their shop. More than half (52.2%) reported that sales would decrease considerably if flavored e-liquids were banned. Controlling for vape shop positions, Table 1 shows the result of the three separate models predicting customer behavior-change intentions. Opposition to a hypothetical ban on non-tobacco e-liquid flavors was associated with opinions that customers would likely not purchase tobacco-flavored e-liquids (b= -0.44, p<0.01), and would likely use smokable tobacco products (b=0.47, p<0.05). Opposition to a hypothetical ban on non-tobacco e-liquid flavors was not significantly associated with opinions that customers would continue to vape (b=0.25, p=0.21).

**Table 1:** A cross-sectional study of perceived impact of a hypothetical ban on non-tobacco flavored e-liquids on customer behavior-change intentions among brick-and-mortar vape shop retailers in the Greater Los Angeles Area, 2020 (N=46)

	*Customer behavior-change intentions*		
	*Model 1 Purchase tobacco flavored e-liquids b (SE) (95% CI)*	*Model 2 Continue to vape b (SE) (95% CI)*	*Model 3 Use smokable tobacco b (SE) (95% CI)*
**Participant’s position in vape shop**			
Manager/clerk	0.65 (0.50) (-0.35–1.64)	0.55 (0.67) (-0.80–1.90)	-0.26 (0.75) (-1.77–1.25)
Owner	0.50 (0.51) (-0.53–1.53)	0.83 (0.70) (-0.57–2.24)	-0.17 (0.78) (-1.74–1.40)
Cashier (Ref.)	-	-	-
**Opposition^†^ to rules such that…**			
Only tobacco flavored e-liquids were allowed at vape shops^§^	-0.44 (0.15)** (-0.75 – -0.12)	0.25 (0.21) (-0.18–0.67)	0.47 (0.24)* (0.00–0.95)

† Higher score means greater opposition: 1 = ‘not at all’, 2 = ‘a little’, 3 = ‘moderately’, and 4 = ‘extremely’.

§ Table 1 was adjusted only for participant vape shop position. Bold values indicate a significant difference at *p<0.05 or **p<0.01.

## DISCUSSION

Compared to ENDS, e-liquids come in more varieties, are cheaper, and are purchased on a more regular basis. To contribute to the evidence base to inform flavor bans, the current cross-sectional study assessed the level of support and perceptions of how customers may react to a hypothetical ban on flavored e-liquids among vape shop retailers in the Greater Los Angeles Area. If a ban on non-tobacco e-liquid flavors were implemented, study participants overwhelmingly reported that they would oppose it. Additionally, most participants reported that if non-tobacco e-liquid flavors were prohibited, vape shop customers would either not purchase tobacco flavored e-liquids or replace vaping with smoking combustible tobacco products – potential unintended consequences that warrant further research.

Study findings align with prior research^17^ regarding policy support and impact of restrictions on ENDS products. Targeted interventions, educational materials, and specific training on the risk of nicotine addiction could potentially gain support for a flavor ban among study participants. Additionally, vape shop retailers’ opinions may resonate with customers and influence attitudes and behaviors. Educating the retailers may lead to comprehensive scientific evidence being communicated to their customers. Future research will need to determine whether retailers and customers discuss a flavor ban, specifically Senate Bill 793^12^, and whether customers perceive retailers as accurate sources of information.

### Limitations

This study has limitations. Data were cross-sectional and longitudinal studies are needed to document how vape shop retailers in the Greater Los Angeles Area adapt to Senate Bill 793^12^. Because data were self-reported, the possibility of recall and social desirability biases exists. Additionally, the sample of 46 brick-and-mortar vape shop retailers in this study might limit generalizability to other geographical areas. Future research should focus on sampling vape shops from different regions of the country and within different policy contexts to compare and contrast retailers’ opinions. Moreover, coupled with the broader contexts in which data collection were carried out [i.e. e-cigarette/vaping-associated lung injury (EVALI) epidemic and COVID-19 pandemic], study findings may not be generalizable to other time periods.

### Implications

Current findings have implications for research and practice. First, given that data were collected before Senate Bill 793, additional research is needed to anticipate the potential impact of the policy on retailers’ attitudes and behaviors, as well as how the policy is implemented and evaluated over time and within different vape shops for compliance and enforcement. In previous research^17^ with licensed commercial tobacco retailers in California, fewer retailers in localities with flavor ordinances had flavored tobacco products available compared to matched jurisdictions without an ordinance, but many still advertised flavored products they could not sell. Indeed, a similar investigation within the context of vape shops and product characteristics is warranted. Moreover, current findings can provide directions for public health professionals and regulatory bodies on the need for stakeholder engagement processes for retailers (e.g. listening sessions).

## CONCLUSIONS

In this cross-sectional study, vape shop retailers in the Greater Los Angeles Area reported that if a ban on non-tobacco e-liquid flavors was enforced, they would oppose it strongly, and that a ban would have a negative impact on their shop (e.g. loss of sales) and customer behavior (e.g. would replace vaping with smoking combustible tobacco products). Future studies are needed to understand what happens in vape shops once policies are in place (e.g. how were the policies communicated to retailers and implemented; are retailers compliant with policies; what happens to product sales). As flavored nicotine and commercial tobacco sales prohibition gains momentum across local jurisdictions, states, and federal government in the US, engaging vape shop retailers may be beneficial in filling research gaps on policy support and impact.

## Data Availability

The data supporting this research are available from the authors on reasonable request.
